# Systematic review and meta-analysis of photon radiotherapy versus proton beam therapy for pediatric rhabdomyosarcoma: TRP-rhabdomyosarcoma 2024

**DOI:** 10.1007/s10147-025-02794-2

**Published:** 2025-06-10

**Authors:** Hiroko Fukushima, Masashi Mizumoto, Sho Hosaka, Yinuo Li, Kazushi Maruo, Yoshiko Oshiro, Hazuki Nitta, Takashi Iizumi, Takashi Saito, Masako Inaba, Ryoko Suzuki, Kei Nakai, Shosei Shimizu, Hideyuki Sakurai

**Affiliations:** 1 Department of Pediatrics, University of Tsukuba Hospital, Tsukuba, Ibaraki 305-8576 Japan; 2 Department of Child Health, Institute of Medicine, University of Tsukuba, Tsukuba, Ibaraki 305-8576 Japan; 3 Department of Radiation Oncology, University of Tsukuba, Tsukuba, Ibaraki 305-8576 Japan; 4 Department of Radiation Oncology, Tsukuba Medical Center Hospital, Tsukuba, Ibaraki 305-8558 Japan; 5 Department of Biostatistics, Institute of Medicine, University of Tsukuba, Tsukuba, Ibaraki 305-8576 Japan; 6 Proton Medical Research Center, University of Tsukuba, 1-1-1 Tennoudai, Tsukuba, Ibaraki 305-8575 Japan

**Keywords:** Rhabdomyosarcoma, Radiotherapy modality, Proton beam therapy, Meta-analysis

## Abstract

**Background:**

Childhood cancer treatment has increasingly achieved favorable long-term survival rates, shifting focus toward reducing long-term comorbidities. Rhabdomyosarcoma (RMS) is the most common pediatric soft tissue tumor, and radiation therapy is essential for its treatment. Proton beam therapy (PBT) is currently utilized due to its potential to reduce long-term complications; however, data on its impact on tumor prognosis remain limited.

**Methods:**

We conducted a meta-analysis to evaluate whether differences in tumor prognosis exist based on radiation therapy modalities, such as photon radiotherapy (photon RT) and PBT, for pediatric parameningial RMS-only study (Group 2) and other pediatric RMS (Group 1). Studies published between 1990 and 2022 were included if they were written in English, included more than 10 cases, and reported outcomes such as overall survival (OS) and local control rates (LC).

**Results:**

A total of 37 results (photon RT: 23, PBT: 14) were analyzed using random-effects meta-analyses. No significant differences were observed between treatment modalities in 1- to 5-year OS (photon RT vs. PBT) in Group 1. Early (1- and 3-year) inferior LC rates were observed with PBT in Group 2.

**Discussion and conclusion:**

Overall survival or LC rates don’t differ significantly between cases treated with photon RT and those treated with PBT. The inferior early LC rates in cases of parameningeal-only RMS study treated with PBT may be attributed to the limited number of studies describing PBT, many of which were single-center reports. Unbiased clinical trial data are needed to clarify differences in early local relapses in parameningeal RMS.

**Supplementary Information:**

The online version contains supplementary material available at 10.1007/s10147-025-02794-2.

## Introduction

Childhood malignancy is a leading cause of childhood mortality in developed countries, making treatment strategies crucial for improving survival outcomes and minimizing long-term comorbidities [1, 2]. Rhabdomyosarcoma (RMS) is the most frequent malignancy among pediatric soft tissue tumors, and its prognosis has improved through a combination of local therapies, such as surgery and radiation therapy, and systemic multi-agent chemotherapy [3, 4]. The disease outcome varies according to the risk group, with high-risk groups showing long-term survival rates of approximately 30–40%, while low-risk groups achieve survival rates exceeding 80% [4–6]. Recurrence in RMS often develops locally, making local treatment strategies highly important. Furthermore, RMS can develop in various parts of the body, leaving several cases unsuitable for adequate surgical resection. For such patients, radiation therapy serves as a key treatment modality. 

Radiation therapy has evolved primarily through advancements in photon radiotherapy (photon RT). However, recent years have seen a rise in the number of patients treated with proton beam therapy (PBT). Despite this, the rarity of RMS, the relatively recent introduction of PBT, and the limited number of treatment facilities mean that information on its tumor control efficacy and long-term side effects remains unclear.

This study aimed to conduct a meta-analysis of existing literature to determine whether differences exist in local control rates and survival outcomes between photon RT and PBT in pediatric RMS patients.

## Methods

### Selection criteria for meta-analysis

The review was conducted in compliance with the Preferred Reporting Items for Systematic Reviews and Meta-Analyses (PRISMA) guidelines and recommendations [7]. Only articles that are written in English were included. All extracted articles were screened by two reviewers. The inclusion criteria were: (1) clinically diagnosed RMS, (2) received radiotherapy (photon RT or PBT), (3) overall survival (OS), or local control (LC) rate with RT can be confirmed in the manuscript, and (4) ≥ 10 cases are specified. 

**Fig. 1:**
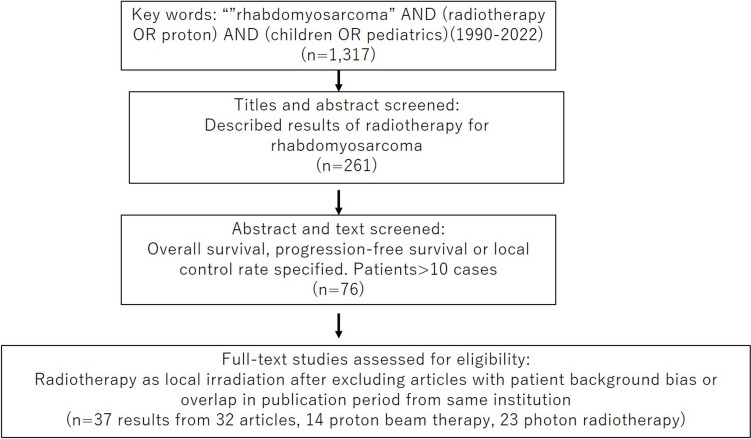
Schematic image of article selection. We conducted a PubMed search using the keywords. We then selected articles that matched our analysis

A PubMed search for “rhabdomyosarcoma” AND (“radiotherapy” OR “proton”) AND (“children” OR “pediatrics”) from 1990 to 2022 identified 1,317 articles. Of these, 193 describing treatment results of RT for RMS were selected based on the title or abstract, and 70 of these articles were found to report OS, PFS or LC rate in the abstract or text. Finally, 37 results from 32 articles (14 PBT, 23 photon RT) were selected based on administration of RT as local irradiation, after exclusion of articles with significant bias in patient background and overlapping publication periods from the same center [6, 8–37]. Where results from different populations were described in a single article, they were counted as two results. The selected articles are shown in Supplementary Table 1. The manuscript selection process is summarized in Fig. 1. Data regarding the authors, year of publication, country, study design, number of patients, deaths, all recurrences, local recurrences, follow-up period, 1- to 5-year OS and LC rates, male-to-female ratio, concurrent chemotherapy rate, embryonal pathological diagnosis rate and treatment modality (photon RT vs. PBT) were collected. If the 1- to 5-year OS and LC rates were not specified in the text, these rates were estimated from the figures. Within the limits specified in the manuscript, the irradiation method of photon RT was either Three-Dimensional Conformal Radiation Therapy (3D-CRT) or Intensity Modulated Radiation Therapy (IMRT). Since radiotherapy plays a more important role in parameningeal RMS where tumors are difficult to completely resect surgically, we conducted this analysis separately for parameningeal-only RMS studies. We assigned studies that excluded parameningeal-only RMS studies to Group 1, which contained 16 results with photon RT and 9 with PBT, respectively [6, 9, 12–15, 17, 19–24, 26, 29, 30, 32, 34–37], and categorized studies describing only parameningeal RMS as belonging to Group 2, which consisted of 7 results with photon RT and 5 with PBT, respectively [8, 10, 11, 16–18, 25, 27, 28, 31, 33].

### Statistical analysis

Random effects meta-analyses of 1- to 5-year OS and LC rates were performed for each modality, and forest plots were drawn. For studies with missing accuracy data, missing values were imputed using the number of cases, risk set size at each year, and mean dropout rate. Heterogeneity in each meta-analysis was evaluated by I-square statistics. Random-effects meta-regression with modality as an explanatory variable were performed for each outcome to compare the modalities. All analyses were performed using R (R Core Team, Vienna, Austria) and its accompanying meta package [38].

## Results

### Main results analysis: Group 1 excluding parameningeal-only RMS studies

**Fig. 2:**
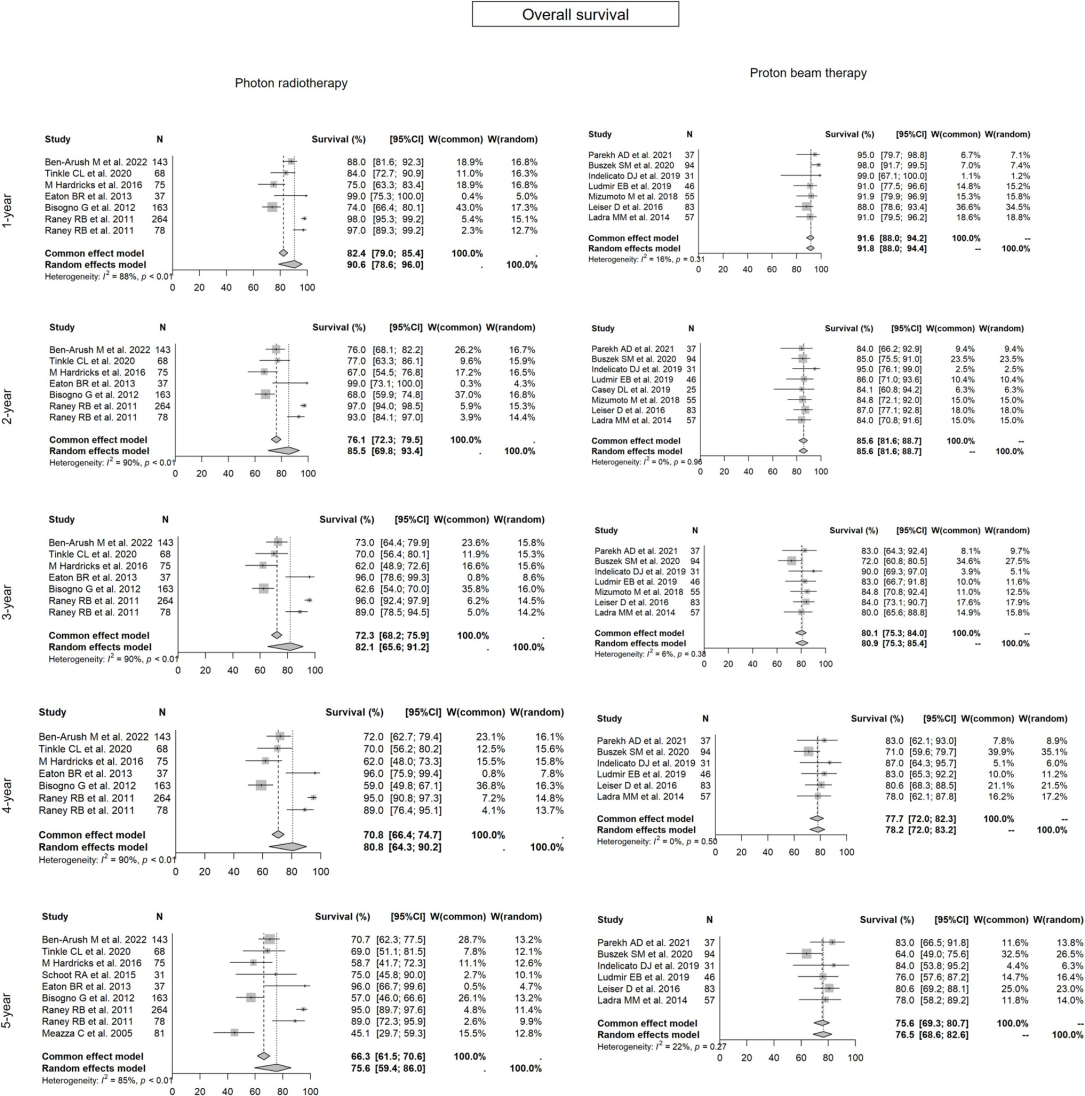
Forest plots of overall survival. Forest plots of 1- to 5-year overall survival for each radiotherapy modality in Group 1. Both modalities gave close results

**Fig. 3:**
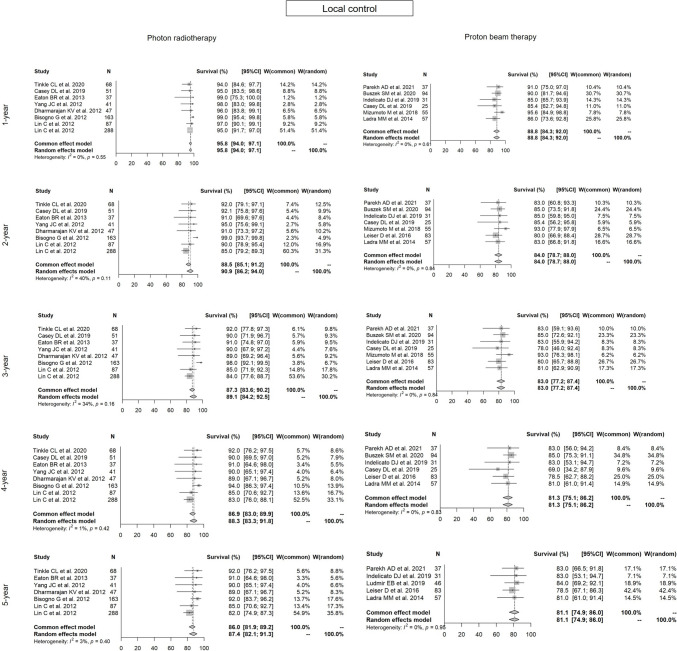
Forest plots of local control. Forest plots of 1- to 5-year local control for each radiotherapy modality in Group 1. Both modalities resulted in close results

Forest plots for each modality for 1- to 5-year OS and LC rates are shown in Figs. 2 and 3, respectively.

A meta-analysis of the 25 selected results from 22 articles (16 for photon RT and 9 for PBT) found 1- to 5-year OS rates (95% confidence interval (CI)) for photon RT vs. PBT of 90.6% (78.6–96.0%) vs. 91.8% (88.0–94.4%) (p = 0.7628); 85.5% (69.8–93.4%) vs. 85.6% (81.6–88.7%) (p = 0.3873); 82.1% (65.6–91.2%) vs. 80.9% (75.3–85.4%) (p = 0.7128); 80.8% (64.3–90.2%) vs. 78.2% (72.0–83.2%) (p = 0.6112); and 75.6% (59.4–86.0%) vs. 76.5% (68.6–82.6%) (p = 0.6141), respectively.

In the meta-analysis, the 1- to 5-year LC rates (95% CI) for photon RT vs. PBT were: 95.8% (94.0–97.1%) vs. 88.8% (84.3–92.0%) (p = 0.1037); 90.9% (86.2–94.0%) vs. 84.0% (78.7–88.0%) (p = 0.1494); 89.1% (84.2–92.5%) vs. 83.0% (77.2–87.4%) (p = 0.1135); 88.3% (83.3–91.8%) vs. 81.3% (75.1–86.2%) (p = 0.1312); and 87.4% (82.1–91.3%) vs. 81.1% (74.9–86.0%) (p = 0.2365), respectively.

Meta-regression analysis was performed using modality (photon RT vs. PBT), male-to-female ratio, concurrent chemotherapy, parameningeal region rate, and embryonal diagnosis rate as risk factors, to the extent such information could be obtained from each article. The actual percentages (median) for each factor (photon RT vs. PBT) were: male-to-female ratio, 37.3–77.4% (55.3%) vs. 43.5–77.4% (54.2%); concurrent chemotherapy, 96.5–100% (100%) vs. 95.8–100% (100%), parameningeal region rate, 0–82.4% (31.8%) vs. 0–68.0% (37.0%); and embryonal diagnosis rate, 0–100% (70.6%) vs. 54.2–96.0% (71.9%), respectively. The total doses of 36 to 54 Gy for photon RT and 36–54 GyE for PBT did not differ significantly and were therefore excluded from our analysis. The meta-regression analysis identified relationships of concurrent chemotherapy and embryonal diagnosis rate with significantly better 2- to 5-year OS (Table 1).

**Table 1:** Meta-regression analysis of potential predictive factors for 1- to 5-year overall survival and local control in Group 1 that excludes studies describing parameningeal-only rhabdomyosarcoma

Factors	Estimate	se	Z val	P val	ci.lb	ci.ub
1-year OS						
Modality of radiotherapy	0.2116	0.7012	0.3018	0.7628	– 1.1627	1.5859
Male-to-female ratio	– 0.0115	0.0341	– 0.3378	0.7355	– 0.0784	0.0554
Chemotherapy rate	– 0.4183	0.3167	– 1.3207	0.1866	– 1.0391	0.2025
Embryonal diagnosis rate	– 0.0337	0.0216	– 1.5613	0.1185	– 0.0760	0.0086
2-year OS						
Modality of radiotherapy	0.2888	0.3341	0.8644	0.3873	– 0.3660	0.9437
Male-to-female ratio	– 0.0146	0.0178	– 0.8193	0.4126	– 0.0494	0.0203
Chemotherapy rate	– 0.3920	0.1246	– 3.1463	0.0017	– 0.6361	– 0.1478**
Embryonal diagnosis rate	– 0.0392	0.0111	– 3.5481	0.0004	– 0.0609	– 0.0176***
3-year OS						
Modality of radiotherapy	– 0.1634	0.4439	– 0.3681	0.7128	– 1.0334	0.7066
Male-to-female ratio	– 0.0139	0.0204	– 0.6805	0.4962	– 0.0539	0.0261
Chemotherapy rate	– 0.4246	0.2122	– 2.0007	0.0454	– 0.8405	– 0.0086*
Embryonal diagnosis rate	– 0.0261	0.0144	– 1.8136	0.0697	– 0.0543	0.0021
4-year OS						
Modality of radiotherapy	– 0.2199	0.4326	– 0.5083	0.6112	– 1.0677	0.6280
Male-to-female ratio	– 0.0049	0.0202	– 0.2429	0.8081	– 0.0444	0.0346
Chemotherapy rate	– 0.4044	0.2021	– 2.0007	0.0454	– 0.8006	– 0.0082*
Embryonal diagnosis rate	– 0.0311	0.0149	– 2.0939	0.0363	– 0.0603	– 0.0020*
5-year OS						
Modality of radiotherapy	0.1961	0.3889	0.5043	0.6141	– 0.5661	0.9583
Male-to-female ratio	– 0.0203	0.0169	– 1.2076	0.2272	– 0.0534	0.0127
Chemotherapy rate	– 0.3540	0.2023	– 1.7496	0.0802	– 0.7506	0.0426
Embryonal diagnosis rate	– 0.0370	0.0155	– 2.3806	0.0173	– 0.0675	– 0.0065*
1-year LC						
Modality of radiotherapy	– 1.1243	0.6910	– 1.6271	0.1037	– 2.4786	0.2300
Male-to-female ratio	– 0.0109	0.0172	– 0.6316	0.5277	– 0.0445	0.0228
Chemotherapy rate	0.0554	0.2314	0.2393	0.8109	– 0.3981	0.5088
Embryonal diagnosis rate	0.0244	0.0282	0.8650	0.3870	– 0.0309	0.0797
2-year LC						
Modality of radiotherapy	– 0.8179	0.5674	– 1.4415	0.1494	– 1.9299	0.2941
Male-to-female ratio	– 0.0188	0.0194	– 0.9705	0.3318	– 0.0569	0.0192
Chemotherapy rate	0.1168	0.1804	0.6475	0.5173	– 0.2368	0.4704
Embryonal diagnosis rate	0.0145	0.0161	0.8955	0.3705	– 0.0172	0.0461
3-year LC						
Modality of radiotherapy	– 0.8436	0.5330	– 1.5827	0.1135	– 1.8882	0.2011
Male-to-female ratio	– 0.0197	0.0188	– 1.0473	0.2950	– 0.0564	0.0171
Chemotherapy rate	– 0.0122	0.1672	– 0.0732	0.9417	– 0.3400	0.3155
Embryonal diagnosis rate	0.0065	0.0163	0.3974	0.6911	– 0.0255	0.0385
4-year LC						
Modality of radiotherapy	– 0.8392	0.5560	– 1.5093	0.1312	– 1.9289	0.2506
Male-to-female ratio	– 0.0071	0.0184	– 0.3847	0.7004	– 0.0431	0.0290
Chemotherapy rate	– 0.1314	0.1705	– 0.7707	0.4409	– 0.4657	0.2028
Embryonal diagnosis rate	0.0020	0.0196	0.1010	0.9195	– 0.0364	0.0404
5-year LC						
Modality of radiotherapy	– 0.5990	0.5060	– 1.1839	0.2365	– 1.5907	0.3927
Male-to-female ratio	– 0.0040	0.0165	– 0.2452	0.8063	– 0.0364	0.0283
Embryonal diagnosis rate	0.0082	0.0159	0.5183	0.6042	– 0.0229	0.0393

P-values: * < 0.05, ** < 0.01, ***p < 0.001

*OS* overall survival; *LC* local control

### Parameningeal tumor subgroup analysis: Group 2

Forest plots for each modality in cases of parameningeal tumor-only for 1- to 5-year OS/LC rates are shown in Supplementary Figs. 2 and 3.

A meta-analysis of the 12 selected results from 11 articles (7 with photon RT and 5 with PBT) found 1- to 5-year OS rates (95% CI) for photon RT vs. PBT of 89.6% (83.7–93.4%) vs. 81.8% (72.8–88.1%) (p = 0.576); 82.4% (79.1–85.3%) vs. 71.6% (60.2–80.3%) (p = 0.296); 76.0% (71.8–79.7%) vs. 66.5% (55.7–75.3%) (p = 0.318); 74.0% (67.4–79.5%) vs. 67.4% (46.2–81.7%) (p = 0.892); and 72.9% (69.0–76.5%) vs. 63.6% (42.1–79.0%) (p = 0.399), respectively.

In the meta-analysis, the 1- to 3-year LC rates (95% CI) for photon RT vs. PBT were: 92.8% (89.3–95.1%) vs. 85.4% (74.8–91.7%) (p = 0.010); 85.1% (82.3–87.5%) vs. 76.8% (63.5–85.8%) (p = 0.229); and 83.5% (80.3–86.3%) vs. 67.9% (50.8–80.1%) (p = 0.028), respectively. Due to the small number of selected articles, 4- and 5-year LC rates could not be evaluated.

Meta-regression analysis was performed using modality (photon RT vs. PBT), concurrent chemotherapy and embryonal diagnosis rate as risk factors, to the extent such information could be obtained from each article. The actual percentages (median) for each factor (photon RT vs. PBT) were: concurrent chemotherapy, 66.7–100% (100%) vs. 100% (100%); and embryonal diagnosis rate, 57.3–88.5% (70.2%) vs. 0–100% (64.7%). The meta-regression analysis identified relationships of modality of radiotherapy with significantly better 1- and 3-year LC in favor of photon RT.

## Discussion

This meta-analysis compared treatment outcomes of photon RT and PBT in pediatric RMS patients. Overall, no significant differences were observed in OS or LC rates between the two modalities. As local recurrence in RMS is frequently observed, and tumor cells can infiltrate surrounding tissues through soft tissue and lymphatic spread, there has been considerable interest in whether PBT, with its limited irradiation area, can provide adequate prevention of local recurrence and/or worse PFS/OS. Our analysis demonstrated non-inferiority of PBT compared to photon RT in terms of OS and LC.

Similar trends were noted in the subgroup analysis focusing on parameningeal RMS cases assigned to Group 2. Proton beam therapy was generally shown to be non-inferior to photon RT regarding OS. However, LC rates at 1 and 2 years were inferior in the PBT group. The fact that only 5 studies describing treatment of parameningeal-only RMS using PBT were available in this meta-analysis may have influenced these findings. Of these studies, one exclusively addressed alveolar RMS [31], another included only cases with significant residual tumors after induction chemotherapy [28], another mentioned that 72% of individuals had tumor diameters > 5 cm at diagnosis [27], and another included 59% of cases with intracranial extension [25]. On balance, these studies predominantly included poor-prognosis groups, which potentially influenced the results. Alveolar RMS is known to harbor *FOXO1* fusion genes in 70–80% of cases, which is associated with a higher risk of recurrence [39, 40]. The inclusion of studies with a high proportion of alveolar RMS in the PBT group may have influenced the outcomes. Similarly, studies focusing on cases with poor chemotherapy response and large tumor diameters (> 5 cm) were likely biased towards poor-prognosis. While intracranial extension is also associated with high local recurrence rates, typically observed in approximately 40% of cases [16], one study in the PBT group reported intracranial extension in approximately 60% of cases, exceeding the typical values [16].

In conclusion, our analysis suggests that radiation therapy modalities, whether photon RT or PBT, do not significantly differ in treating RMS. However, there is limited evidence regarding LC rates in parameningeal RMS, making definitive conclusions challenging. Accumulating long-term data on comorbidities and tumor outcomes is essential for objective evaluations in the future.

## Supplementary Information

Below is the link to the electronic supplementary material.

Supplementary file1 (XLSX 26 KB)

Supplementary Figure 1. Forest plots of overall survival in the parameningeal-only studies. Forest plots of 1- to 5-year overall survival for each radiotherapy modality in Group 2. Both modalities gave close results. Supplementary file2 (JPG 902 KB)

Supplementary Figure 2. Forest plots of local control in the parameningeal-only studies. Forest plots of 1- to 5-year local control for each radiotherapy modality in Group 2. Early local control at 1 and 3 years is inferior for proton beam therapy. Not enough studies were selected to describe forest plots of 4- and 5-year local control for proton beam therapy. Supplementary file3 (JPG 892 KB)

## Data Availability

All data generated or analyzed during this study are included in this article. Further enquiries can be directed to the corresponding author.

## Abbreviations

LC: Local control
OS: Overall survival
PBT: Proton beam therapy
RT: Radiotherapy
RMS: Rhabdomyosarcoma
CI: Confidence interval
TRP: Tsukuba review project
