# Supplementary figures and images for: Systematic review and meta-analysis of photon radiotherapy versus proton beam therapy for pediatric rhabdomyosarcoma: TRP-rhabdomyosarcoma 2024
Source: Int J Clin Oncol. 2025 Jun 10;30(8):1670–7. doi: 10.1007/s10147-025-02794-2 (PMC12296975; doi:10.1007/s10147-025-02794-2)

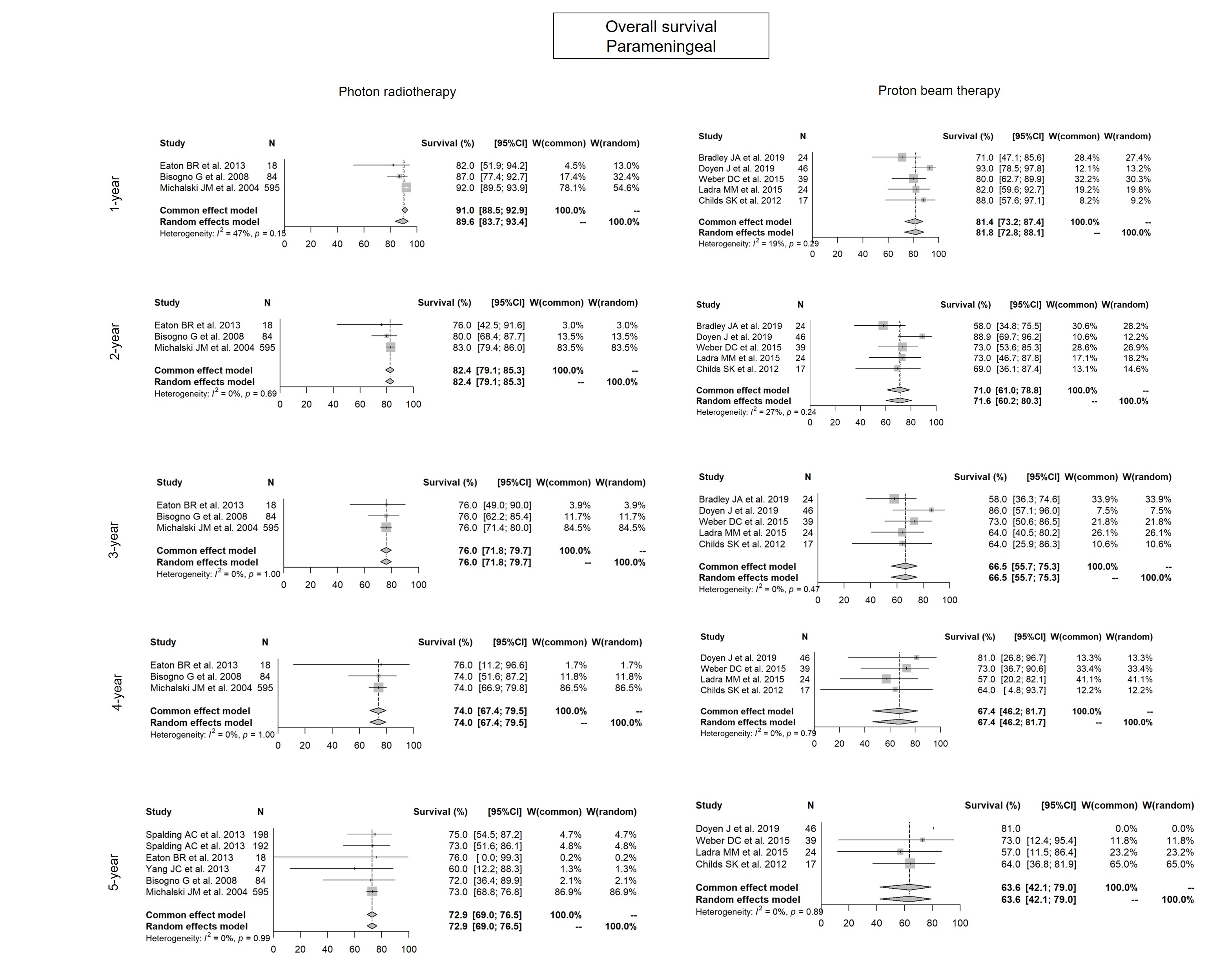

Supplement: Supplementary file 2 — Supplementary Figure 1. Forest plots of overall survival in the parameningeal-only studies. Forest plots of 1- to 5-year overall survival for each radiotherapy modality in Group 2. Both modalities gave close results. Supplementary file2 (JPG 902 KB) [file 10147_2025_2794_MOESM2_ESM.jpg]

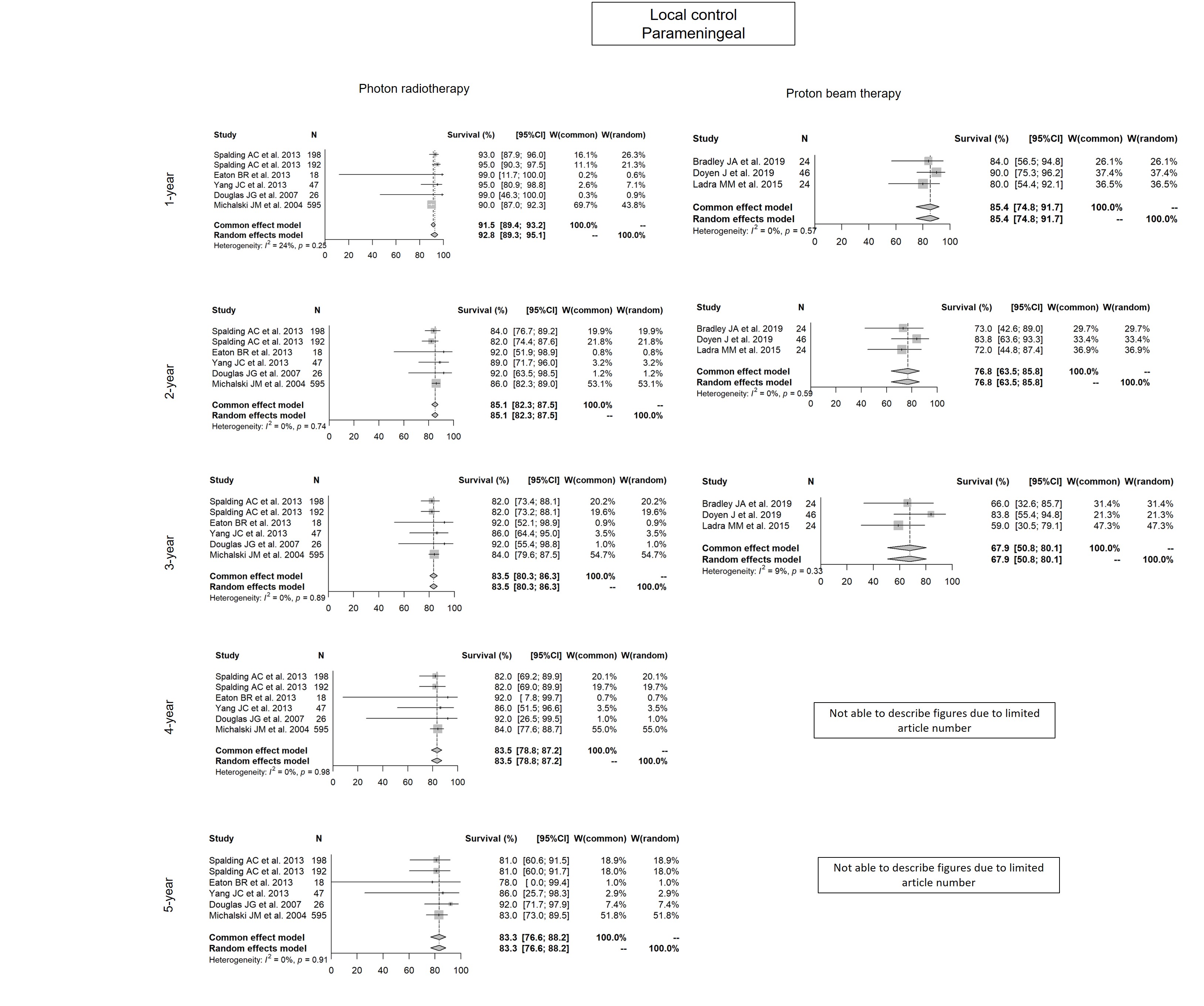

Supplement: Supplementary file 3 — Supplementary Figure 2. Forest plots of local control in the parameningeal-only studies. Forest plots of 1- to 5-year local control for each radiotherapy modality in Group 2. Early local control at 1 and 3 years is inferior for proton beam therapy. Not enough studies were selected to describe forest plots of 4- and 5-year local control for proton beam therapy. Supplementary file3 (JPG 892 KB) [file 10147_2025_2794_MOESM3_ESM.jpg]
